# Characterizing Pharmacokinetics in Children With Obesity—Physiological, Drug, Patient, and Methodological Considerations

**DOI:** 10.3389/fphar.2022.818726

**Published:** 2022-03-10

**Authors:** Jacqueline G. Gerhart, Stephen Balevic, Jaydeep Sinha, Eliana M. Perrin, Jian Wang, Andrea N. Edginton, Daniel Gonzalez

**Affiliations:** ^1^ Division of Pharmacotherapy and Experimental Therapeutics, UNC Eshelman School of Pharmacy, The University of North Carolina at Chapel Hill, Chapel Hill, NC, United States; ^2^ Department of Pediatrics, Duke University Medical Center, Durham, NC, United States; ^3^ Duke Clinical Research Institute, Durham, NC, United States; ^4^ Department of Pediatrics, UNC School of Medicine, The University of North Carolina at Chapel Hill, Chapel Hill, NC, United States; ^5^ Department of Pediatrics, Johns Hopkins University Schools of Medicine and School of Nursing, Baltimore, MD, United States; ^6^ Office of Drug Evaluation IV, Center for Drug Evaluation and Research, US Food and Drug Administration, Silver Spring, MD, United States; ^7^ School of Pharmacy, University of Waterloo, Waterloo, ON, Canada

**Keywords:** obesity, pediatrics, drug development, pharmacokinetics, physiologically-based pharmacokinetics

## Abstract

Childhood obesity is an alarming public health problem. The pediatric obesity rate has quadrupled in the past 30 years, and currently nearly 20% of United States children and 9% of children worldwide are classified as obese. Drug distribution and elimination processes, which determine drug exposure (and thus dosing), can vary significantly between patients with and without obesity. Obesity-related physiological changes, such as increased tissue volume and perfusion, altered blood protein concentrations, and tissue composition can greatly affect a drug’s volume of distribution, which might necessitate adjustment in loading doses. Obesity-related changes in the drug eliminating organs, such as altered enzyme activity in the liver and glomerular filtration rate, can affect the rate of drug elimination, which may warrant an adjustment in the maintenance dosing rate. Although weight-based dosing (i.e., in mg/kg) is commonly practiced in pediatrics, choice of the right body size metric (e.g., total body weight, lean body weight, body surface area, etc.) for dosing children with obesity still remains a question. To address this gap, the interplay between obesity-related physiological changes (e.g., altered organ size, composition, and function), and drug-specific properties (e.g., lipophilicity and elimination pathway) needs to be characterized in a quantitative framework. Additionally, methodological considerations, such as adequate sample size and optimal sampling scheme, should also be considered to ensure accurate and precise top-down covariate selection, particularly when designing opportunistic studies in pediatric drug development. Further factors affecting dosing, including existing dosing recommendations, target therapeutic ranges, dose capping, and formulations constraints, are also important to consider when undergoing dose selection for children with obesity. Opportunities to bridge the dosing knowledge gap in children with obesity include modeling and simulating techniques (i.e., population pharmacokinetic and physiologically-based pharmacokinetic [PBPK] modeling), opportunistic clinical data, and real world data. In this review, key considerations related to physiology, drug parameters, patient factors, and methodology that need to be accounted for while studying the influence of obesity on pharmacokinetics in children are highlighted and discussed. Future studies will need to leverage these modeling opportunities to better describe drug exposure in children with obesity as the childhood obesity epidemic continues.

## Introduction

Almost 20% of children in the United States (US) and 10% of children worldwide are currently classified as obese (World Health Organization, 2016; NCD-RisC, 2017; Skinner et al., 2018). The prevalence of pediatric obesity is growing, with obesity rates quadrupling in children in the US in the past quarter-century. This is an imperative public health issue, as children with obesity are at increased risk of developing comorbidities, such as cardiovascular disease and type 2 diabetes (Skinner et al., 2015). This means that children with obesity require more prescription drugs than those without obesity, and children with obesity often experience worse outcomes with clinically-used dosing (Scherrer et al., 2015; Solmi and Morris, 2015) In adults, obesity is defined as an abnormally high body size for a given height, measured by body mass index (BMI). A generally accepted reference BMI limit is 30 kg/m^2^, beyond which an adult will be classified as obese. Such a fixed reference body size metric does not apply to children because of the baseline changes in the body size to height ratio due to continuous growth and development (Kuczmarski et al., 2000). In children, BMI overall increases naturally as the child matures, and also undulates, particularly in the age from 3–8 years when there is a physiological “BMI dip” (Kuczmarski et al., 2000). Therefore, to characterize childhood obesity, an age- and sex-specific reference BMI limit is generally considered. The US Centers for Disease Control and Prevention (CDC) has recommended the 95th percentile of the BMI-to-age curve as the reference limit, with a BMI percentile ≥95 but <120% of the 95th percentile indicating Class 1 obesity, a BMI from 120–140% of the 95th percentile indicating Class II obesity, and a BMI >140% of the 95th percentile indicating Class III obesity (Gulati et al., 2012). Many additional body size measures have been proposed to measure obesity, which are summarized in Table 1. While these body size measures are often more accurate in describing fat and lean body size in children, they can be more challenging to calculate in a clinical setting (Freedman and Sherry, 2009; Anderson and Holford, 2017; Sinha et al., 2018; Green et al., 2020).

**TABLE 1:** Selected direct and indirect measures of body size for children.

**Total body weight (TBW)**
TBW [kg]=Measured weight of child [kg]
**Body mass index (BMI)**
$BMI\;\left[\frac{kg}{m^{2}}\right]=\frac{TBW\;\left[kg\right]}{Height\;{\left[m\right]}^{2}}$
**Body surface area (BSA)^a^**
$BSA\;\left[m^{2}\right]=0.024265*TBW\;{\left[kg\right]}^{0.5378}*Height\;{\left[cm\right]}^{0.3964}$
**Ideal body weight (IBW)^b^**
$IBW\;\left[kg\right]=BMI_{50}\left[\frac{kg}{m^{2}}\right]*Height\;{\left[m\right]}^{2}$
$BMI_{50}\left(boys\right)\;\left[\frac{kg}{m^{2}}\right]=24.27-\frac{8.91}{1+{\left(\frac{Age\;\left[y\right]}{15.78}\right)}^{4.40}}$
$BMI_{50}\left(girls\right)\;\left[\frac{kg}{m^{2}}\right]=22.82-\frac{7.51}{1+{\left(\frac{Age\;\left[y\right]}{13.46}\right)}^{4.44}}$
**Fat-free mass (FFM)^c^**
$FFM\left(boys\right)\;\left[kg\right]=\left[0.88+\left(\frac{1-0.88}{1+{\left(\frac{Age\;\left[y\right]}{13.4}\right)}^{-12.7}}\right)\right]\left[\frac{9270*TBW\;\left[kg\right]}{6680+\left(216*BMI\;\left[\frac{kg}{m^{2}}\right]\right)}\right]$
$FFM\left(girls\right)\;\left[kg\right]=\left[1.11+\left(\frac{1-1.11}{1+{\left(\frac{Age\;\left[y\right]}{7.1}\right)}^{-1.1}}\right)\right]\left[\frac{9270*TBW\;\left[kg\right]}{8780+\left(244*BMI\;\left[\frac{kg}{m^{2}}\right]\right)}\right]$

**Body fat percent (BFP)^d^**
$BFP\left(boys\right)\;\left[\%\right]=\frac{0.647*0.0598*e^{2.050*BMI_{95}\;\left[\frac{kg}{m^{2}}\right]}}{0.647+0.0598*\left(e^{2.050*BMI_{95}\;\left[\frac{kg}{m^{2}}\right]}-1\right)}+0.1140-0.00890*Age\;\left[y\right]$
$BFP\left(girls\right)\;\left[\%\right]=\frac{1.080*0.1930*e^{0.897*BMI_{95}\;\left[\frac{kg}{m^{2}}\right]}}{1.080+0.1930*\left(e^{0.897*BMI_{95}\;\left[\frac{kg}{m^{2}}\right]}-1\right)}-0.0856+0.00682*Age\;\left[y\right]$


a See (Haycock et al., 1978), *n* = 81 subjects.

b See (Pai and Paloucek, 2000; Callaghan and Walker, 2015), *n* = 108 subjects.

c See (Janmahasatian et al., 2005; Al-Sallami et al., 2015), *n* = 1,011 subjects.

d See (Green et al., 2020), *n* = 4,274 subjects.

Unfortunately, despite the frequent use of prescription drugs in children with obesity, the data to inform their specific dosing is lacking (Solmi and Morris, 2015). While US legislation in recent years has increased the amount of pediatric data submitted to the FDA, this has not bridged the gap in studies conducted in children with obesity (Harskamp-van Ginkel et al., 2015; Mulugeta et al., 2016; Sun et al., 2017). Although data for four drugs submitted to the FDA emphasized the effect of body size on pharmacokinetics (PK) in children, only one FDA label to date provides dosing information in children with obesity (Vaughns et al., 2018; Cleocin Phosphate, 2020). Currently, the best body size descriptor to use and whether to cap dosing is unknown for many drugs dosed in patients with obesity. Thus, there is a lack of standard dosing practice, with clinicians using different body weight measures or simply capping at the adult recommended dose. Many ethical and logistical barriers to conducting clinical PK studies in children with obesity contribute to this data gap. Pharmacokinetic studies in general traditionally require intensive sampling to adequately characterize drug disposition to determine dosing that is both safe and efficacious, which is not always feasible in pediatric populations. A stigma surrounding obesity can depress enrollment rates of these children. Lower enrollment of children with obesity relative to those without obesity means that they are often underrepresented in all-comer trials, and that these trials may thus be underpowered to detect differences in exposure between children with and without obesity. The additional enrollment time and trial cost might hugely limit the inclusion of the full age and body size range of children required to characterize drug disposition in children with obesity.

**FIGURE 1:**
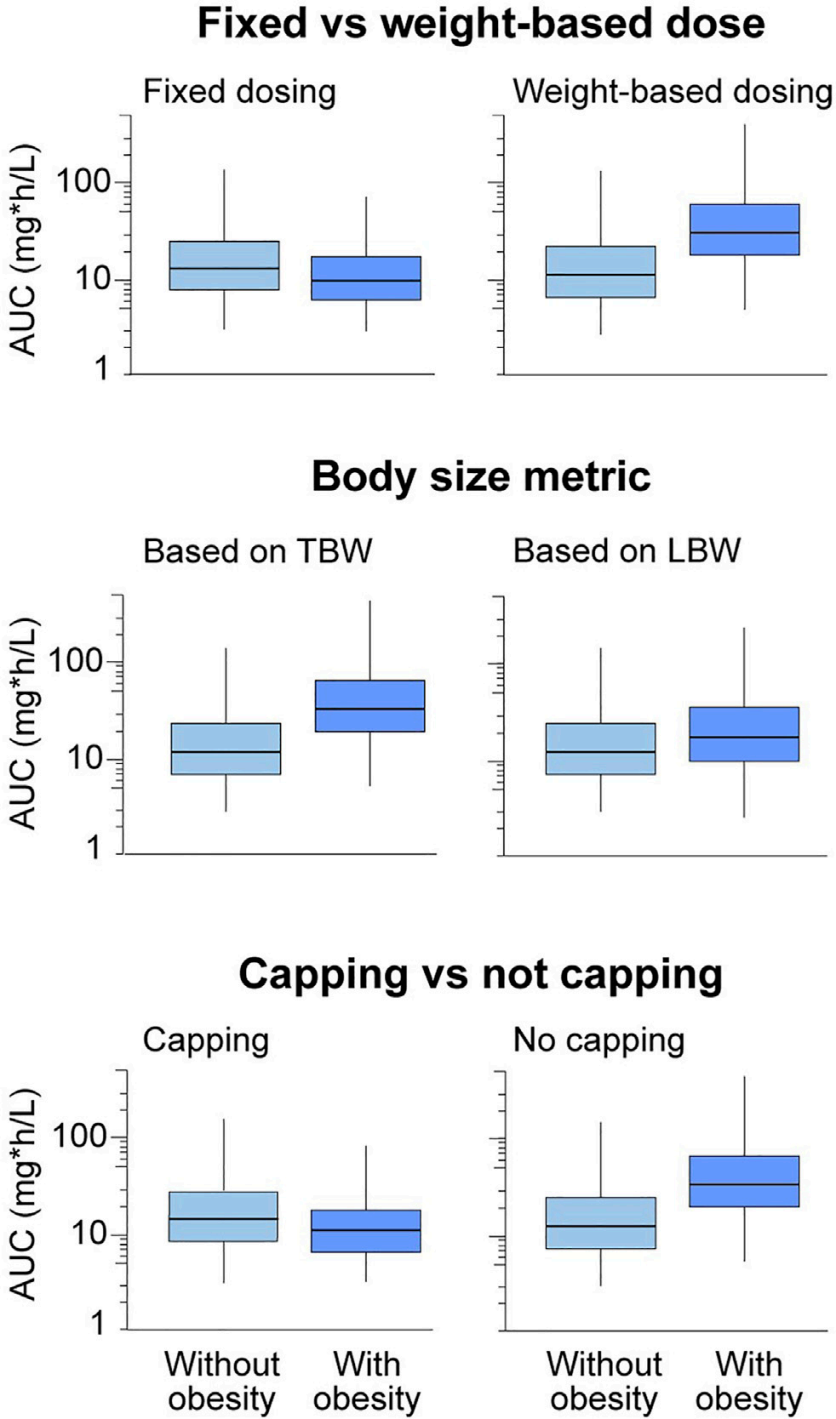
Trends in drug exposure in children with versus without obesity will depend on different types of dosing considerations, including fixed versus weight-based dosing, total body weight versus a lean body weight size descriptor, and capping versus not capping. Boxplots show relative drug exposure in children with versus without obesity for a theoretical drug under these various dosing scenarios with hypothetical drug exposure. Note that these are expected trends with obesity for different dosing constraints, and the magnitude of the change may vary depending on drug properties. Additional constraints on dosing include available formulations and dosing routes, as well as previously established therapeutic ranges. AUC, area under the plasma concentration-versus-time curve; LBW, lean body weight; TBW, total body weight.

Many dosing considerations contribute to confusion around dosing in children with obesity (Figure 1). Dosing guidance in children with and without obesity is typically bucketed into subgroups by age, though the age bounds on these subgroups may differ by drug. Typically used age subgroups include 2—< 6 years, 6—< 12 years, 12—< 18 years, and 18—< 21 years describing early childhood, middle childhood, early adolescence, and late adolescence, respectively (Williams et al., 2012; U. S. Food and Drug Administration, 2014). PK may differ between these pediatric age subgroups due to differences in growth and maturation, or obesity onset and the physiologically healthy BMI variations within these subgroups. However, these age classifications might not fit neatly within a particular drug’s indication, or enrollment challenges may preclude subgrouping. Most pediatric clinically used dosing is weight-based (i.e., using total body weight or a measure of lean body weight), but may vary in terms of what body size metric is used to calculate the absolute dose. Fixed dosing can also be considered, particularly if the drug is dosed primarily in older pediatric populations or has a wide therapeutic window. Dose capping, or implementing a maximum total dose, is common in children who receive weight-based dosing. Often, the adult recommended dose or dose cap is also used in children with obesity, as is best clinical practice in the absence of further dosing guidance in these children. The Medication Dosing in Overweight and Obese Children report issued by the Pediatric Pharmacy Advocacy Group states that the regular adult dose for any particular drug should be considered for children with obesity exceeding 40 kg in total body weight (Matson et al., 2017). It is not recommended to exceed the recommended adult maximum dose in these children (Matson et al., 2017). However, extrapolating dosing guidance from adults, with or without obesity, is not scientifically advised given that differences in PK may exist due to maturation or obesity disease progression. The reality of how a drug is currently formulated (e.g., formulation route, fixed co-administration ratio, or pre-filled syringes) may constrain actual use of the ideal dosing. In this case, it is important to consider how a drug realistically be dosed in clinical practice.

**FIGURE 2:**
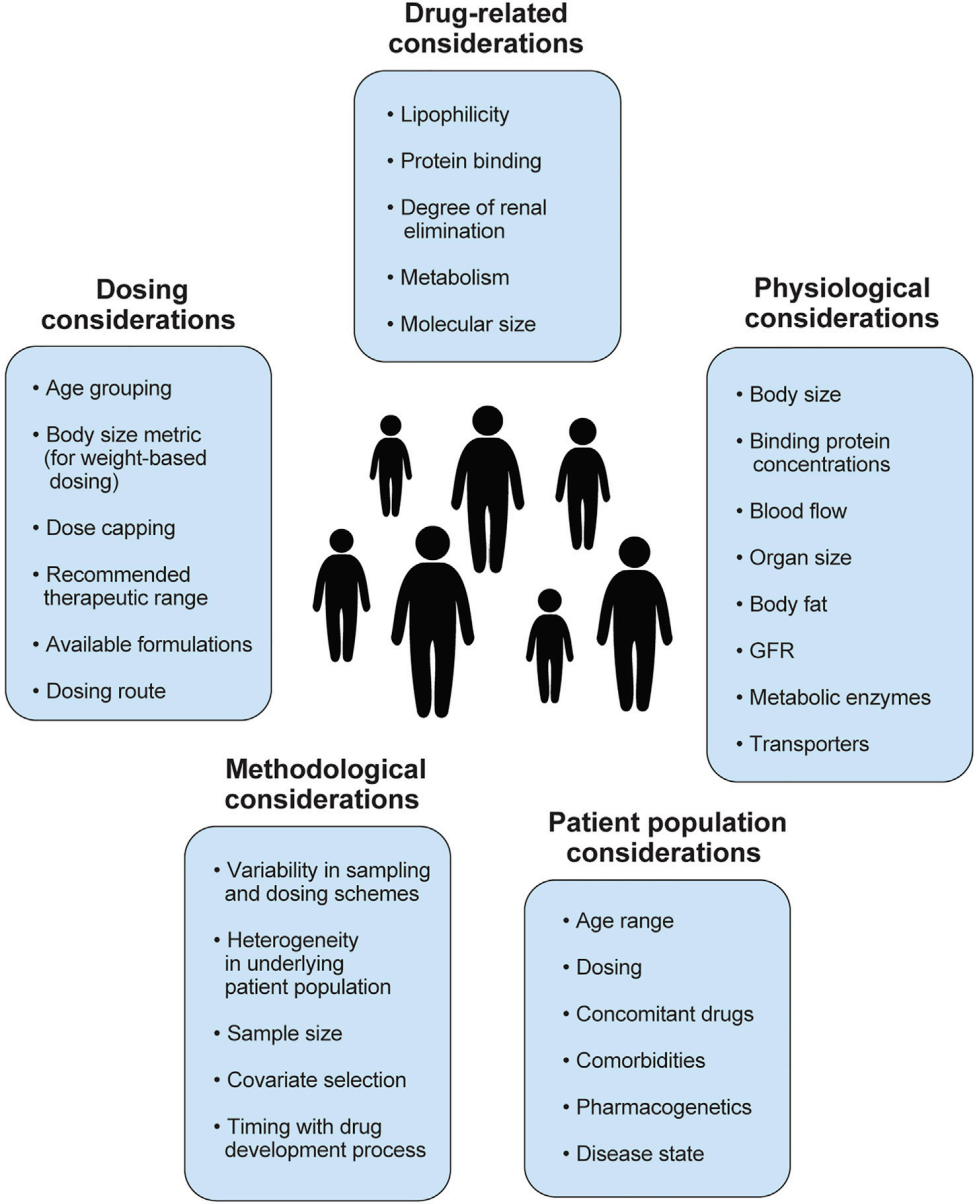
Summary of factors to consider when studying pharmacokinetics in children with obesity, including physiological, drug-related, patient population, methodological considerations. Several factors to consider in dose selection are also included. GFR, glomerular filtration rate.

In order to evaluate and choose appropriate doses for children with obesity, it is necessary to understand the PK drivers of dosing, including clearance and volume of distribution. Specifically, studying the change in PK of drugs in children with versus without obesity is imperative. By altering body size and composition, obesity can also influence drug disposition that already has a baseline influence from age-related growth and development. One prior review found that 65% of drugs studied in children, including those with obesity, demonstrated altered PK with obesity (Harskamp-van Ginkel et al., 2015). However, none of these drugs have dosing guidance that would account for altered body size and composition with obesity (Harskamp-van Ginkel et al., 2015). By mechanism, the PK differences are the combined effect of obesity-related structural and functional changes in physiology (e.g., organ size, composition, and function) and the drug-related properties (e.g., physicochemical and absorption, distribution, metabolism, and elimination [ADME] properties). Therefore, the effect of obesity on drug disposition depends on the particular drug in question, which needs separate assessment. This also highlights the fact that a universal dosing scheme for obesity is unlikely to exist. Instead, it should be developed based on separate evaluations for obesity’s effect on a particular drug’s disposition. In reality, characterizing these effects of obesity is not straightforward in children. First, unlike adults, pediatric PK studies are conducted in patient populations, which potentially confounds the effect of obesity by other pathological influences. Further, because of methodological constraints, pediatric PK studies are often suboptimal to characterize these effects accurately and precisely. Therefore, apart from the drug-related properties, understanding the patient-related and methodology-related aspects are also equally important to consider while elucidating the effect of obesity in children. In this review, we explore how these four considerations—physiology, drug parameters, patient population characteristics, and methodological considerations (Figure 2)—can impact the assessment of PK in children with obesity. We conclude by exploring opportunities to bridge the dosing knowledge gap in these children.

## Physiological Considerations

The following equation can characterize the impact of various factors on PK parameters:

$$PK_{i}=PK_{standard}*f_{size}*f_{function}*f_{age}$$

where *PK*_*i*_ is an individual’s PK parameter, *PK*_*standard*_ is the typical value of the PK parameter, and *f*_*size*_, *f*_*function*_, and *f*_*age*_ are the effects of body size, organ function, and age (i.e., growth and maturation) on the PK parameter, respectively (Anderson and Holford, 2009). For children with obesity, the effects of both increased body size and age are important for characterizing PK. In this section, we consider the physiological effect of obesity as the effect of increased body size and fat mass alone, rather than pathological changes in renal or hepatic function, for example.

Many physiological variables that directly influence the volume and composition of plasma and tissues compartments can impact a drug’s volume of distribution (V_ss_) during obesity. V_ss_ can be defined using the equation below:

$$V_{ss}=V_{plasma}+V_{tissue}*\frac{f_{u,p}}{f_{u,t}}$$

where *V*_*plasma*_ is plasma volume, *V*_*tissue*_ is tissue water volume, *f*_*u,p*_ is fraction unbound in plasma, and *f*_*u,t*_ is fraction unbound in tissue. Increased *V*_*plasma*_ and *V*_*tissue*_ (given more distribution space) can increase cardiac output during obesity (Vasan, 2003; Colles et al., 2006; Gerhart et al., 2021), potentially increasing the V_ss_ on an absolute scale. However, the extent of increase in V_ss_ is also dependent on the drug binding to plasma protein (that determines $f_{u,p}$ ) as well as to the tissue components (that determines $f_{u,t}$ ), which are potentially altered due to altered composition during obesity. While studies have shown that plasma composition with respect to serum albumin and hematocrit are largely unaffected by obesity, it changes with respect to *α*1-acid glycoprotein (AAG), which increases approximately 2-fold with obesity in adults (Benedek et al., 1983, 1984; Blouin et al., 1987; Gerhart et al., 2021). However, this increase in AAG has not yet been identified in pediatric populations with obesity due to limited studies (Gerhart et al., 2021).

**FIGURE 3:**
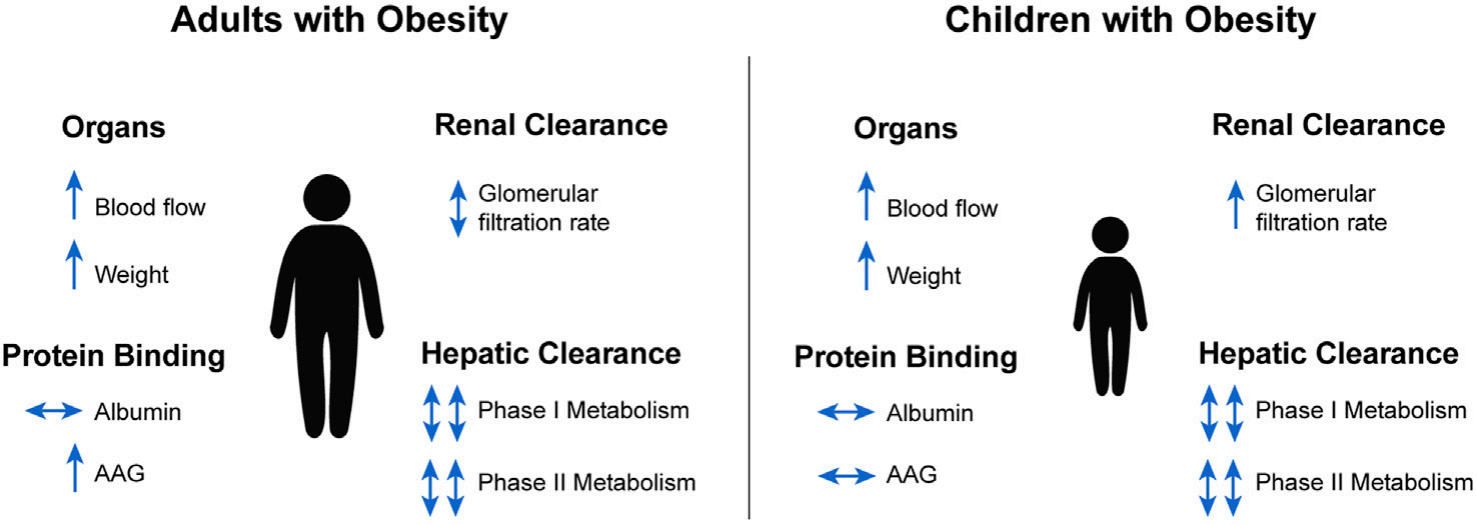
Summary of obesity-induced physiological changes relevant to pharmacokinetics in adults and children (Ghobadi et al., 2011; Gerhart et al., 2021). AAG, alpha-1 acid glycoprotein.

An increase in body fat with obesity is also accompanied by an increase in lean mass to provide additional structural (e.g., increased skeletal strength) and functional (e.g., increased metabolic need) support due to extra weight gain from adiposity. For example, key clearance organs such as the kidney and liver increase 19% and 18% in mass on average, respectively, with obesity (Nawaratne et al., 1998; Gerhart et al., 2021). These organ mass increases were determined by a series of magnetic resonance imaging and dual x-ray absorptiometry studies in adults and ultrasound scans in pediatric populations with and without obesity (Hwaung et al., 2019; Gerhart et al., 2021). Unlike body fat, these increases in non-fat organs do not increase proportionately with obesity. As a result, both the lean mass and body fat fractions (percent of total body weight) can be different between two children with the same body weight, but one with and one without obesity. Such alteration in body composition (i.e., the relative content of fat and non-fat tissues) between the two children may cause their individual V_ss_ to differ, especially for drugs that do not uniformly distribute into the fat and non-fat tissues (i.e., drugs with varying lipophilicities). See Figure 3 for a summary of observed obesity-induced physiological changes relevant to PK for adults and children with obesity.

In general, the rate-limiting physiological variables of drug elimination do not change proportionately with increasing body size associated with obesity, raising concerns about the applicability of the conventional mg/kg dosing with obesity (Nawaratne et al., 1998; Young et al., 2009). For hepatic clearance, the key variables are functional liver size, hepatic blood flow, and activity and abundance of drug-metabolizing enzymes (DMEs). Liver size and blood flow are both increased with obesity, as mentioned above. Inflammatory cytokines such as interleukin 6, often associated with obesity, have been shown to down-regulate activity of cytochrome P450 (CYP) enzymes and hepatic drug transporters in mice and humans (Richardson and Morgan, 2005; Schmitt et al., 2011; Cayot et al., 2014; Morgan et al., 2018; Abualsunun et al., 2020). There is additional evidence of some obesity-associated alterations in activity of DMEs and transporters in adult clinical studies. Evidence was available mainly for CYP3A4 and CYP2E1, where up to a 40% decrease and a 140% increase in DME activity was reported (Ulvestad et al., 2013; Krogstad et al., 2021). However, such evidence is lacking in children with obesity, as it is challenging to obtain biopsy samples from these children. There are very few clinical studies reported in children that extrapolate metabolic enzyme activity from drug clearance or metabolite formation rate. For example, a pediatric study of the CYP3A4 substrate midazolam reported a 38% increase in absolute clearance with obesity, possibly conflicting with adult reports due to lower comorbidity rates in pediatric obesity (van Rongen et al., 2018). There is even less evidence to inform potential changes in transporter activity in children with obesity. While some studies of adults with nonalcoholic steatohepatitis, a common obesity-related fibro-inflammatory disease of the liver, show altered transport by organic anion transporting polypeptide (OATP) and multidrug resistance-associated protein (MRP), such investigations have yet to be explored in adults or children with obesity specifically (Pierre et al., 2017; Ali et al., 2018; Sjöstedt et al., 2021).

For renal clearance, key variables include glomerular filtration rate (GFR) and tubular secretion and reabsorption. While absolute GFR is 12–29% higher in children 2–18 years of age with obesity, the more typically reported body surface area-normalized GFR is 1–3% lower on average (Correia-Costa et al., 2016; Gerhart et al., 2021). Increases in kidney size and GFR can impact the clearance of drugs with significant renal elimination. Changes in tubular reabsorption and secretion have not yet been explored in children with obesity. However, studies of drugs that are preferentially reabsorbed (e.g., lithium) and secreted (e.g., procainimide, ciprofloxacin, and cimetidine) in the renal tubule in adults suggest both increased tubular reabsorption and secretion with obesity (Christoff et al., 1983; Reiss et al., 1994; Blouin and Warren, 1999). Decreased renal clearance of these drugs supports the theory that tubular reabsorption and secretion is altered in these cases (Christoff et al., 1983; Reiss et al., 1994; Blouin and Warren, 1999).

## Drug-Related Considerations

### Physicochemical Properties

The volume of distribution is important because it influences the selection of loading doses. Comparing the weight-normalized volume of distribution using total versus lean body weight measures helps illustrate how obesity impacts this PK parameter. If total body weight-normalized volume of distribution is similar between children with and without obesity, this suggests that the drug experiences high distribution into the additional fat mass. If total body weight-normalized volume of distribution is less in children with obesity, then there is not full distribution of the drug into excess fat mass. See Table 2 for a selection of drugs previously studied in children with obesity. One review evaluating this across several prior studies found that total body weight was the best metric for characterizing volume of distribution in adults with obesity (for 40% of drugs). In contrast, lean body weight was the best metric for clearance (for 35% of drugs) (Green and Duffull, 2004). This meta-analysis also found that total body weight dosing for moderate to high lipophilic drugs best described the data empirically, whereas lean body weight-based dosing best described low lipophilic drugs (Green and Duffull, 2004). A similar empirical analysis is lacking for children with obesity. However, no single body size metric has been found to describe the overall impact of obesity on these PK parameters, as the best metric varies depending on the drug under study. Note that while drug concentration in the blood is easily measured, actual sites of distribution cannot be determined without directly sampling various tissues. Thus, it is challenging to evaluate changes in tissue distribution of a drug with obesity.

**TABLE 2:** Representative sample of reported pharmacokinetic changes with obesity for drugs dosed in children.

Drug	Patient population	Sample size	Age	Body size	Dosing	PK conclusions	Dosing conclusion
Acetaminophen Barshop et al. (2011)	Case-control study of children with NAFLD	Without NAFLD: *n* = 12 boys	Without NAFLD: 14.4 (4.5) years	Without NAFLD: 26.22 (10.95) kg/m^2^ BMI; 1.21 (1.42) BMI z-score	Single 5 mg/kg oral dose capped at 325 mg	Children with NAFLD had higher concentrations of the glucuronide metabolite but no significant differences in PK parameters	---
		With NAFLD: *n* = 12 boys	With NAFLD: 14.8 (1.8) years	With NAFLD: 34.00 (6.14) kg/m^2^ BMI; 2.30 (0.43) BMI z-score			
Busulfan Browning et al. (2011)	Children undergoing hematopoietic stem cell transplant conditioning	BMI percentile <25%: *n* = 17 (25.0%)	Mean 7.1 (6.1) [0–21] years	30.6 [2.5–117.8] kg TBW	BMI percentile <25%: 3.6 (0.7) mg/kg IV	Children with high BMIs had higher AUCs after TBW dosing compared to children with mid-range or low BMIs. 53% of children with high BMIs would have AUCs ≥20% outside the target using AIBW dosing	Children with higher BMIs require a lower dose (2.9 mg/kg TBW) to match AUC to children with mid-range (4.0 mg/kg TBW) or low (3.6 mg/kg TBW) BMIs. Therapeutic drug monitoring is recommended
		25-<85%: *n* = 29 (42.6%)			25-<85%: 4.0 (1.1) mg/kg IV		
		≥85%: *n* = 22 (32.4%)			≥85%: 2.9 (1.1) mg/kg IV; based on TBW		
Clindamycin Smith et al. (2017)	Children receiving drug per standard of care	420 total PK samples from 220 children (76 with obesity)	[range 0.01–20.5] years	BMI percentile <95%: *n* = 144 (65.4%)	Drug dosed per standard of care	Obesity status did not explain inter-individual variability after accounting for TBW in PK parameters	Results support TBW-based dosing for all children
				≥95–99%: *n* = 46 (20.0%)			
				>99%: *n* = 30 (13.6%)			
Doxorubicin Thompson et al. (2009)	Children with cancer	22 children (6 with body fat >30%)	15 [3.3–21.5] years	51.5 [12.4–80] kg TBW	Any infusion <24 h on 1,2 days schedule not based on IBW or capped	Doxorubinol, but not doxorubicin, clearance was lower in patients with body fat >30%	---
				19.7 [13.2–30.0] kg/m^2^ BMI			
				25 [15–36] body fat %			
Fentanyl Maharaj et al. (2021)	Children receiving drug per standard of care	53 samples from 32 children (31 with obesity)	13 [2–19] years	52 [16–164] kg TBW	Drug dosed per standard of care	The risk of achieving C_ss_ values above the target increased with increasing body weight. Use of a theoretical allometric relationship between weight and CL described the PK in children with obesity	A proposed model-derived continuous infusion strategy based on TBW maximized the probability of achieving the target C_ss_ range
Gentamicin Choi et al. (2011)	Case-control study of children with and without obesity	25 children without obesity and 25 children with obesity	[2–18] years	---	Without obesity: 2.25 (0.41) mg/kg TBW	Children with obesity had significantly higher peak and trough concentrations despite receiving significantly lower mg/kg TBW doses	Empirical dose reduction and therapeutic drug monitoring is necessary for children with obesity
					With obesity: 1.86 (0.43) mg/kg TBW		
Midazolam Gade et al. (2020)	---	67 adolescents (36 with obesity) with 13 plasma samples each	Without obesity: 14 [11–17] years	Without obesity: 55 [33–76] kg TBW	Single 1 μg IV bolus microdose	Faster inter-compartmental CL and a greater peripheral V_d_ were observed in adolescents with obesity	Current dosing guidelines using TBW may lead to supra- or sub-therapeutic dosing in adolescents with obesity
			With obesity: 14 [11–17] years	With obesity: 77 [46–124] kg TBW			
Midazolam van Rongen et al. (2015)	Adolescents undergoing surgery	19 children with obesity or who were overweight (BMI percentile ≥85%)	Mean 15.9 [12.5–18.9] years	Mean 102.7 [62–149.8] kg TBW	Either 2 or 3 mg IV	TBW did not influence CL but did affect peripheral V_d_. This was explained by excess weight rather than maturational growth	Results suggest a potential need for higher initial infusion rates in adolescents with obesity
				Mean 36.1 [24.8–55.0] kg/m^2^ BMI			
Vancomycin Le et al. (2015)	Case-control study of children with and without obesity	87 matched pairs with 389 total plasma samples	Without obesity: 10.0 [IQR 4.8–15.2] years	Without obesity: 44.0 [IQR 23.4–78.1] kg TBW	Without obesity: mean 47.4 (13.0) [IQR 39.9–53.3] mg/kg/d TBW	TBW and allometric weight were reasonable estimations of differences in CL and V_d_	PK differences are small and not likely clinically relevant in dose variation
			With obesity: 10.2 [IQR 4.5–14.8] years	With obesity: 31.3 [IQR 16.8–47.1] kg TBW	With obesity: mean 41.9 (12.0) [IQR 33.4–50.1] mg/kg/d TBW		
Vancomycin Nassar et al. (2015)	Case-control study of children with and without obesity	77 peak and trough concentrations from 51 children	5 [0.5–18] years	17.6 [3.5–83.0] kg TBW; Children were divided into underweight, normal weight, and overweight groups	20 mg/kg TBW IV BID	PK parameters for all weight groups were similar	---

Values reported as mean (standard deviation) or median [range] unless otherwise specified.

AIBW, adjusted ideal body weight; AUC, area under the plasma concentration-versus-time curve; BID, twice daily; BMI, body mass index; CL, clearance; C_ss_, steady-state plasma concentration; IBW, ideal body weight; IQR, interquartile range; IV, intravenous; NAFLD, nonalcoholic fatty liver disease; PK, pharmacokinetic; TBW, total body weight; V_d_, volume of distribution.

### Elimination Routes

The degree to which a drug is eliminated via renal excretion (e.g., by GFR) versus hepatic metabolism can also impact the degree to which the drug’s clearance is altered with obesity. Vancomycin is a commonly used drug cleared entirely by GFR. Two studies evaluating vancomycin clearance differences in age-matched children with or without obesity observed a decrease (∼25% or less) in weight-normalized clearance with obesity, with the magnitude of the difference declining in younger age groups (Le et al., 2015; Nassar et al., 2015). This is in-line with assumptions based on physiological changes, which suggest that absolute GFR increases only 12–29% on average with obesity, not proportionally with increased total body weight with obesity (Correia-Costa et al., 2016; Gerhart et al., 2021).

A drug’s metabolism profile can also make a drug more susceptible to changes with obesity depending on which DMEs are responsible. For example, one pediatric study of chlorzoxazone, a CYP2E1 substrate, found that overall systemic clearance normalized to weight was significantly higher in children with versus without obesity, while the renal elimination remained unchanged (Gade et al., 2018). This suggests a potential increase in CYP2E1 activity and/or expression in children with obesity that drove two-fold higher absolute clearance (Gade et al., 2018). Studies of CYP3A substrates are mixed. Weight-normalized clearance decreased with obesity in children receiving clindamycin and in one study of midazolam, but was similar or slightly elevated relative to children without obesity in a study of fentanyl and two other midazolam studies (Harskamp-van Ginkel et al., 2015; van Rongen et al., 2015; Gade et al., 2020; Gerhart et al., 2021; Maharaj et al., 2021). This is likely also influenced by the degree to which liver blood flow impacts the hepatic clearance of these drugs based on their differing extraction ratios, or possibly differences in relative affinity for CYP3A5, 3A4, and/or 3A7. This also contrasts with midazolam studies in adults with obesity, which suggest a decrease in CYP3A4 metabolism (van Rongen et al., 2018). More studies are needed to confirm reduced CYP3A metabolism observed in adults with obesity. While these changes in drugs with a primary elimination route can be straightforward, these elimination-driven changes might be less clear for drugs with mixed elimination pathways.

### Biologics

Compared to small molecule drugs, biologics have several unique considerations with respect to drug absorption, distribution, metabolism, and excretion. Although biologics represent a heterogeneous drug class, including vaccines and blood products, we will specifically focus on therapeutic proteins.

Biologics are characterized by complex quaternary structures and very large molecular weights (1.3–251 kDa), which are susceptible to degradation in the gastrointestinal tract, and thus not suitable for oral administration (Vugmeyster et al., 2012; Meibohm, 2019). Accordingly, all currently used biologics are given parenterally, either intravenously, subcutaneously, or intramuscularly. Biologics generally have volumes of distribution that approximate plasma volume, although molecular size, charge, and the presence of certain components (e.g., Fc fragments) can impact the drug’s volume (Vugmeyster et al., 2012). Following subcutaneous or intramuscular administration, small biologics (<1 kDa) diffuse readily into blood, whereas large proteins generally reach systemic circulation through convective transport into lymphatic vessels. However, monoclonal antibodies (mAbs) or other drugs with an Fc component can undergo transcellular transport to the systemic circulation (Meibohm, 2019). Lastly, drug clearance for biologics can occur via multiple mechanisms, including proteolysis, intracellular catabolism through the reticuloendothelial system, or target-mediated drug disposition (TMDD) by binding to therapeutic targets (Vugmeyster et al., 2012). As a result, some biologics (e.g., mAbs) often show both linear and non-linear elimination processes. In addition, some patients may develop anti-drug antibodies against therapeutic proteins, often accelerating drug clearance (Mould, 2015).

The impact of obesity on the PK of biologics has not been extensively studied, but population pharmacokinetic (PopPK) modeling in adults suggests that body weight is a significant covariate on PK parameters for patients with and without obesity for many biologics. Using the most commonly prescribed biologics as an example, larger body weight is associated with higher clearance for etanercept, rituximab, and adalimumab; and higher volume of distribution for adalimumab and infliximab (Lee et al., 2003; Fasanmade et al., 2009; Nader et al., 2017; Rozman et al., 2017). Moreover, the impact of body size on biologics PK may be one potential mechanism that explains why patients with rheumatic diseases and with obesity have a substantially higher risk of failing treatment with anti-cytokine biologics (Singh et al., 2018).

Although the mechanisms by which biologics PK is altered by body size are not fully understood, several theories have been proposed. First, subcutaneous blood flow is reduced in individuals with obesity, potentially reducing or delaying the absorption of therapeutic proteins administered subcutaneously (Frayn and Karpe, 2014). Second, adipose tissue may have reduced expression of the neonatal Fc receptor (FcRn), which is responsible for recycling mAbs and other biologics with an Fc fragment (Hodkinson, 2017). Third, proteolytic clearance is higher with body weight (Mould, 2015). And lastly, obesity results in a state of chronic inflammation through increased expression of multiple inflammatory cytokines (Kern et al., 2001; Gremese et al., 2014; Schmidt et al., 2015). The inflammatory state could potentially increase biologics drug clearance through either increased protein catabolism or elevation of the baseline level of the target cytokine itself, causing enhanced TMDD. For example, both high pre-treatment C-reactive protein (CRP) and tumor necrosis factor alpha (TNFα) levels (i.e., target baseline) are inversely correlated with infliximab trough levels (Wolbink et al., 2005; Takeuchi et al., 2011). Moreover, infliximab’s half-life decreases from 14 to 8 days when CRP increases from 0.1 mg/L to 14 mg/L (Ternant et al., 2013).

Until the impact of obesity on biologics PK is better understood, it is difficult to provide definitive guidance on dosage adjustment for this drug class. However, because total blood volume relative to body size is reduced in patients with obesity, intravenous biologics that are dosed on a mg/kg basis (i.e., intravenous immune globulin [IVIG]) could potentially result in higher plasma concentrations when absolute body weight is used (Hodkinson, 2017). Accordingly, IVIG is often dosed using adjusted or ideal body weight (Anderson and Olson, 2015; Ameratunga, 2017). Conversely, differences in drug exposure were not different when subcutaneous immune-globulin was administered in patients with and without obesity, underscoring the heterogeneous effect that obesity may have on drug PK depending on the drug, route of administration, and possibly other unobserved patient characteristics (Shapiro, 2013).

## Patient Population Considerations

Due to physiologic BMI undulations, physiologic developmental body fat changes, and other developmental changes, children over a wide range of ages (i.e., children ≥2 years of age through adulthood) should be enrolled to fully understand changes in PK. Unlike adults, for children the effect of obesity on PK can vary with age groups since age itself has a baseline influence on PK. This means that the difference in PK between children with and without obesity at one age group may be different within another age group (particularly after the onset of adolescence), and extrapolation without accounting for the age effect may lead to bias (van Rongen et al., 2018). For example, in a PK study of vancomycin in 87 age-matched pairs of children with and without obesity, weight-normalized clearance was similar between these two groups for children 2–12 years of age. In contrast, weight-normalized clearance decreased with obesity for those >12 years (Le et al., 2015). This observation may reflect the magnitude of the difference not being large enough to be detected by the study’s sample size, or perhaps because a longer duration of obesity in older children leads to more pronounced obesity-induced changes or different BMI changes (fat-free mass, etc.) amongst these age groups.

Patient-related factors other than age can also affect PK and drug exposure, such as differences in dosing (e.g., weight-based dosing using different body size measures or drug formulations), concomitant drug administration, or pharmacogenetic variation in DMEs and transporters. Pediatric PK trials are commonly done by opportunistic sampling during standard of care treatment. Therefore, co-administration of certain drugs (e.g., enzyme-inducing antiepileptic drugs) interacting with an elimination pathway (e.g., CYP metabolism) of the drug in question potentially confounds the impact of obesity on clearance. The same confounding effect can come from pharmacogenetic alteration (i.e., gain or loss of function) in DMEs and transporters. Unfortunately, drug-drug interaction and pharmacogenetic studies are less commonly conducted in children, and these effects (if present) would potentially confound the assessment of obesity on PK (Gonzalez and Sinha, 2021). Obesity-focused pediatric trials should consider such potential effects of these patient-related factors (beyond just age) during trial design.

Further, an inherent issue with studying populations with obesity is the presence of comorbidities affecting PK, potentially confounding the influence of obesity (although these comorbidities are usually less prevalent in children than in adults with obesity). The common comorbidities associated with pediatric obesity include prediabetes and diabetes mellitus, dyslipidemia, prehypertension and hypertension, non-alcoholic fatty liver disease (NAFLD), polycystic ovary syndrome (PCOS), obstructive sleep apnea, and psychiatric conditions (Styne et al., 2017). NAFLD, which is estimated to occur in 38% of US children with obesity, has been shown to alter transport and clearance of hepatically eliminated drugs in adults (Schwimmer et al., 2006; Merrell and Cherrington, 2011). Reductions in kidney function with prolonged duration of diabetes, for example, may also impair drug clearance. These factors can change the fraction eliminated by a given elimination route, resulting in different obesity-induced changes. Conversely, for drugs studied for indications primarily in populations with obesity (e.g., metformin), there may be limited subjects without obesity to fully evaluate pharmacokinetic trends across a range of body sizes.

## Methodological Considerations

Typical PK studies in adults with obesity often involve a case-control matched noncompartmental analysis (NCA), in which PK parameters are calculated from individual concentration-versus-time profiles. However, the rich sampling scheme required to generate these profiles is difficult to collect in children, for whom generally only sparse samples are available. Even if rich pediatric sampling is available for children with obesity, it is still challenging to use NCA outside of a phase I study in the face of PK confounders. Due to the nature of pediatric studies, there is often significant inter-patient variability around sampling (including number and timing of sampling) and dosing regimens in addition to patient heterogeneity in terms of age, disease state, organ function, etc., which precludes the use of a naïve-pooled approach for PK parameter estimation.

Modeling and simulations tools can greatly aid understanding of PK in children with obesity and were recommended for consideration in all pediatric drug development programs by the FDA’s Advisory Committee for Pharmaceutical Science and Clinical Pharmacology in 2012 (Food and Drug Administration, 2019). PopPK models are commonly used in PK analysis of drugs in children owing to sparse sampling requirements (Tremoulet et al., 2014; Standing et al., 2018). PopPK is a useful modeling tool because it can be used to analyze even sparse real-world data and assess the effect of different body size metrics on PK parameters. However, PopPK is very data-driven and heavily reliant on study design. To evaluate the effect of obesity status (or age or organ function), data from a full age, size, or organ function range is required, and thus a robust study design is needed. In PopPK models, allometric scaling using plausible body size metrics (e.g., total body weight or fat-free mass) should be explored instead of a fixed *a priori* scaling with total body weight raised to the power of 0.75 (Sinha et al., 2019). An additional effect of age on clearance and volume of distribution (i.e., separate from body size) should also be tested (Germovsek et al., 2019). Simulations of target PK metrics (e.g., AUC or steady-state trough concentration) from the PopPK model should be used to find optimal dosing scalars (e.g., total body weight) and dosing regimens that would achieve equivalent exposure in children with and without obesity for a given age group.

Conversely, physiologically-based pharmacokinetic (PBPK) modeling is a bottom-up PK modeling approach that does not require extensive data, and thus it is not reliant on the PopPK constraints mentioned above. PBPK models account for changes in physiology and body composition in children to accurately guide dosing in children with obesity, all while requiring minimal data to develop. These models integrate physiological parameters (i.e., organ size and blood flow), drug parameters (i.e., physicochemical properties and metabolism), and known efficacy targets to describe drug disposition mechanistically and inform dosing (Cao and Jusko, 2012; Kuepfer et al., 2016). PBPK models offer advantages over traditional methods, such as PopPK models, by 1) describing developmental and physiological changes in children to capture the effect of age and body size on disposition, 2) providing prediction of concentrations in any tissue to allow for assessment of drug disposition at the target site, and 3) incorporating mechanistic information that is required to understand differences in PK. However, because of their foundations in physiology, these PBPK models require significant physiological information on the population under study, some of which is still unknown in children with obesity. This bottom-up approach can also require *in vitro* and physicochemical information not yet available, particularly for newer drugs under study. Nevertheless, PBPK modeling represents a useful tool for simulating PK and exposure of drugs dosed in children with obesity, even in the face of little data.

## Opportunities and Future Directions

A better understanding of the effect of obesity on PK for drugs commonly dosed in children with obesity is an urgent public health need, particularly as the already high prevalence of obesity in children grows. While the PK of many drugs has been shown to be altered in both adults and children as described herein, the impact of excess body size on drugs’ clearance and volume of distribution has not been determined. To improve the safety and efficacy of drugs dosed in children, clinical PK studies need to enroll a more representative cohort of children across a wide range of body sizes. While there are numerous ethical and logistical constraints to be overcome in the enrollment of children with obesity, there are opportunities to bridge this PK data gap. This is supported by a new regulatory requirement for clinical trials to consider diversity and inclusion, including children with obesity.

Sparse sampling typical in pediatric PK studies may be supplemented with opportunistic or electronic health record (EHR) data. Opportunistic data involves collecting data from routine laboratory blood draws from pediatric patients receiving a particular drug under study per standard of care. Further clinical data documented in a pediatric patient’s chart, such as demographics (e.g., age, BMI) and laboratory values (e.g., albumin, serum creatinine), can be easily collected. Similarly, drug concentration and dosing information from therapeutically monitored drugs, such as enoxaparin, can be collected retrospectively from EHR data for PK analysis (Richard et al., 2013). Consent rates for these types of data collection are typically higher, as it minimizes risk to the pediatric patient. However, opportunistic and EHR data have more inherent variability, putting additional pressure on sample sizes. Additionally, EHR data collection will only be feasible for the few drugs that undergo therapeutic drug monitoring.

PBPK modeling has tremendous potential for bottom-up prediction of PK in a target population without the need for data. Therefore, in the absence of data in this special population of children with obesity, PBPK modeling should be applied to complement PopPK analysis, especially in finding the likely effective dose for children with obesity. This approach has been previously used in adults with obesity to successfully predict drug clearance of eight drugs, including alprazolam, caffeine, chlorzoxazone, cyclosporine, midazolam, phenytoin, theophylline, and triazolam (Ghobadi et al., 2011). This approach has also recently been used to predict clearance and volume of distribution differences in children with versus without obesity receiving clindamycin and trimethoprim/sulfamethoxazole (Gerhart et al., 2021). There is an opportunity to continue applying this PBPK modeling approach to other drugs while evaluating the virtual population’s underlying assumptions as new data becomes available about the physiological changes altered by obesity in children.

With the rise in popularity of biologics, there is an urgent need to address existing knowledge gaps in the optimal use of these drugs in children, particularly those with obesity. For example, researchers are still investigating the precise mechanisms that govern biologics drug disposition, such as anti-drug antibody-mediated clearance, factors affecting absorption, and the role of FcRn on biologics distribution, among others (Vugmeyster et al., 2012). Moreover, there is an ongoing need to understand the impact of genetic polymorphisms that affect both disposition processes (e.g., mutations in FcRn) and drug response (e.g., mutations in the TNFα receptor) (Jančić et al., 2015; Billiet et al., 2016). Accordingly, it will be increasingly important to support translational pharmacokinetic/pharmacodynamic (PK/PD) studies for modeling and simulation. By leveraging data from these mechanistic PK/PD studies, PBPK models were successfully scaled from adults to children for several biologics, including infliximab, palivizumab, and bevacizumab (Malik and Edginton, 2019; Basu et al., 2020). Lastly, clinical studies are needed to identify optimal target concentrations before the promise of PBPK model-guided dosing becomes a reality in children with obesity (Balevic and Sagcal-Gironella, 2022).

**FIGURE 4:**
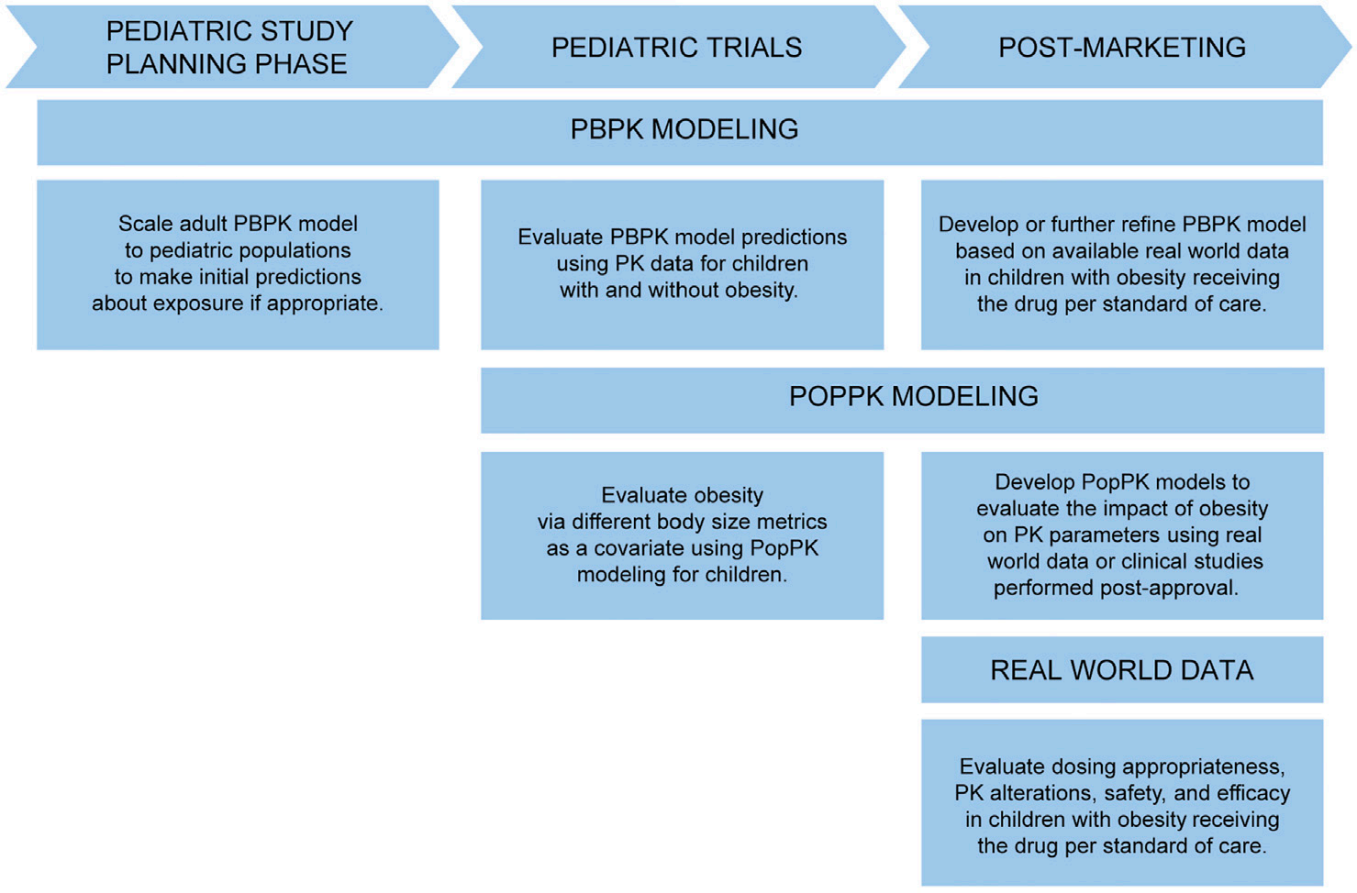
Approaches to evaluate PK in children with obesity throughout pediatric drug development and into the post-marketing phase. PBPK, physiologically-based pharmacokinetic; PK, pharmacokinetics; PopPK, population pharmacokinetic.

Ultimately, considering PK in children with obesity should be a part of pediatric drug development and considered as early in the pediatric drug development process as possible. This is especially true when the drug under study is particularly likely to be indicated in children with obesity (e.g., a diabetes or statin drug). PBPK modeling can be used at the beginning of a pediatric drug development program as part of the pediatric investigation plan (PIP) to estimate PK changes and inform dosing in children with versus without obesity *a priori*, then evaluated prospectively in pediatric studies (Figure 4). In this way, PBPK is used to make initial predictions until pediatric data becomes available. When these pediatric studies are performed, the impact of body size on PK can be further evaluated using PopPK modeling. If a drug is already approved, investigating dosing in children with obesity using opportunistic and EHR data can occur.

## Conclusion

Children with obesity are a rapidly growing patient population with a large knowledge gap in PK and dosing. Studies of children with obesity must consider obesity-induced changes in physiology relevant to PK, such as increased organ size and elimination mechanisms. Drug properties, such as lipophilicity or elimination route, can impact the degree to which these obesity-induced changes affect PK. Additional patient factors, particularly age range, concomitant drug administration, and comorbidities, must also be considered for a particular drug under study. Methodological factors like variability in sampling and dosing schemes and the underlying patient population and sample size must be accounted for when studying PK in children with obesity.

Children with obesity are susceptible to altered PK due to obesity-related physiological changes, such as increased organ size and drug elimination capacity. The extent to which obesity affects PK depends on the drug properties, such as lipophilicity and elimination pathway(s). However, several other factors that are mainly related to patient population, such as age group, concomitant drug administration, and comorbidities, can confound obesity-related changes in PK. Methodological constraints in pediatric trials like limited sample size and sparse sampling scheme impose further challenges in characterizing PK changes in children with obesity. This review has highlighted the key considerations related to physiology, drug parameters, patient factors, and methodology that need to be accounted for while studying the influence of obesity on PK in children. A well-designed study and appropriate use of modeling and simulation techniques can ensure appropriate dosing in children with obesity, thereby delivering safe and effective therapies to this vulnerable group of patients.
